# Regulation of COX2 Expression in Mouse Mammary Tumor Cells Controls Bone Metastasis and PGE2-Induction of Regulatory T Cell Migration

**DOI:** 10.1371/journal.pone.0046342

**Published:** 2012-09-28

**Authors:** John Karavitis, Laura M. Hix, Yihui H. Shi, Rachael F. Schultz, Khashayarsha Khazaie, Ming Zhang

**Affiliations:** 1 Departments of Molecular Pharmacology and Biological Chemistry, Northwestern University Feinberg School of Medicine, Chicago, Illinois, United States of America; 2 Robert H. Lurie Comprehensive Cancer Center, Northwestern University Feinberg School of Medicine, Chicago, Illinois, United States of America; Baylor College of Medicine, United States of America

## Abstract

**Background:**

The targeting of the immune system through immunotherapies to prevent tumor tolerance and immune suppression are at the front lines of breast cancer treatment and research. Human and laboratory studies have attributed breast cancer progression and metastasis to secondary organs such as the bone, to a number of factors, including elevated levels of prostaglandin E2 (PGE2) and the enzyme responsible for its production, cyclooxygenase 2 (COX2). Due to the strong connection of COX2 with immune function, we focused on understanding how variance in COX2 expression manipulates the immune profile in a syngeneic, and immune-competent, mouse model of breast cancer. Though there have been correlative findings linking elevated levels of COX2 and Tregs in other cancer models, we sought to elucidate the mechanisms by which these immuno-suppressive cells are recruited to breast tumor and the means by which they promote tumor tolerance.

**Methodology/Principal Findings:**

To elucidate the mechanisms by which exacerbated COX2 expression potentiates metastasis we genetically manipulated non-metastatic mammary tumor cells (TM40D) to over-express COX2 (TM40D-COX2). Over-expression of COX2 in this mouse breast cancer model resulted in an increase in bone metastasis (an observation that was ablated following suppression of COX2 expression) in addition to an exacerbated Treg recruitment in the primary tumor. Interestingly, other immune-suppressive leukocytes, such as myeloid derived suppressor cells, were not altered in the primary tumor or the circulation. Elevated levels of PGE2 by tumor cells can directly recruit CD4+CD25+ cells through interactions with their EP2 and/or EP4 receptors, an effect that was blocked using anti-PGE2 antibody. Furthermore, increased Treg recruitment to the primary tumor contributed to the greater levels of apoptotic CD8+ T cells in the TM40D-COX2 tumors.

**Conclusion/Significance:**

Due to the systemic effects of COX2 inhibitors, we propose targeting specific EP receptors as therapeutic interventions to breast cancer progression.

## Introduction

Treatment of breast cancer has greatly improved patient morbidity and mortality, though these current standards of treatment still allow nearly 25% of patients to succumb to the disease [1]. This underscores the necessity for improved treatment strategies that limit toxicity and achieve lasting tumor regression. The idea of one's immune system surveying tumors was first suggested by Paul Ehrlich in 1909 [2]. Since then, the field of tumor immunology has sought to realize those therapeutic goals by harnessing the immune system to eliminate the body's own cancerous cells. In contrast to this, a tumor can also manipulate the immune system to create an environment that promotes its growth, a process referred to as immuno-editing. Approaches to inhibit a tumors ability to hijack and utilize the immune system to remain undetected are very appealing therapeutic potentials still in their infancy.

Initially, transformed cells divide into a growing tumor that eventually disrupts the surrounding stroma, triggering release of pro-inflammatory signals that recruit mediators of the innate immune system [3]. These cells have limited direct killing ability through various methods [4], [5]. Immature dendritic cells are also recruited to the site, where they engulf necrotic and apoptotic tumor cells and present tumor-associated antigen (TAA) epitopes on MHC class II receptors to naïve CD4+ T cells [6]. This activates CD4+ naïve T cells that in turn release inflammatory cytokines, stimulating naïve CD8+ T cells to clonally expand into TAA-specific cytotoxic T lymphocytes (CTLs) [7]. The activated TAA-specific CD4+ helper T cells and CTLs amass to the primary tumor site, where tumor-specific CTLs recognize and eliminate antigen-presenting tumor cells through secretion of perforin and induction of Fas/FasL-mediated apoptosis, while unknowingly selecting for less immunogenic tumor cells [8].

An important subset of CD4+ T cells known as regulatory T cells (Tregs), are instrumental in the induction and maintenance of normal peripheral tolerance and prevention of autoimmunity [9]. Tregs play a central role in immunosuppression by directly inhibiting the function of many cells, including CD8+ T cells [10]. They suppress effector cells mainly through contact-dependent mechanisms, although Treg secretion of transforming growth factor-β (TGF-β) and IL-10 have also been shown to inhibit tumor-specific CTL cytotoxicity *in vivo* [11]. Tregs can suppress proliferation of activated effector T cells by direct contact and induce transcriptional down-regulation of the proliferative cytokine IL-2, inhibiting their clonal expansion [12]. Additionally, Tregs can induce direct killing of effector cells through release of granzyme and perforin [13], [14]. Notably, Tregs can function to suppress many of the host defenses utilized to prevent cancer proliferation and progression, making Treg recruitment by developing tumors a critical step in evasion of the immune response and tumor cell survival.

Numerous clinical studies and animal cancer models demonstrate that tumors are able to recruit Tregs, and this is associated with advanced disease progression in gastric cancer, leukemia, non-small cell lung cancer, and breast cancer [15]. Specifically, in human breast cancer patients, the percentage of Tregs at the tumor site is positively correlated with disease progression from normal to ductal carcinoma in situ (DCIS), and from DCIS to invasive carcinoma [16]. Despite the correlation of Treg accumulation and advanced cancer progression, the mechanisms by which Tregs induce progression of the tumor remain unclear. To this end, tumors are known to secrete high levels of TGF-ß, which has been shown *in vitro* to convert naïve T cells to Tregs [17]. In addition to TGF-ß, cyclooxygenase 2 (COX2), as well as its main product, prostaglandin E2 (PGE2) have also been found to stimulate *de novo* Treg conversion from naïve CD4+ T cells [18]. Interestingly, elevated expression of both COX2 and PGE2 have been demonstrated at the tumor site, with high levels of COX2 expression being associated with highly aggressive tumors [19]. However, though reports have correlated enhanced COX2 expression with increased levels of Tregs in breast cancer, there is no data providing evidence of the mechanism by which this occurs.

In this study, we provide evidence that over-expressing COX2 (TM40D-COX2), and subsequently elevated level of PGE2 in a low-aggressive breast TM40D cancer cell line, increases the rate of bone metastasis, comparable to a highly metastatic TM40D-MB breast cancer line. In contrast, bone metastasis in the mammary tumor cell line that does not express COX2, TM40D-MB-shCOX2, was lost compared to the high-COX2 expressing lines (TM40D-COX2 and TM40D-MB). *In vitro* proliferation and *in vivo* tumor growth rates were not affected, suggesting PGE2-induced metastasis is not linked to a varied proliferation rate. Additionally, we show that COX2 over-expression in TM40D tumors alters their immune profile from a high infiltration of antitumor CD4^+^ T helper cells, to a high tumor frequency of suppressive CD4^+^ FoxP3^+^ Tregs. Enriched Tregs preferentially are recruited to the factors released in the supernatant of TM40D-COX2 and TM40D-MB cells, an effect that can be inhibited using an antibody against PGE2. Moreover, we show that COX2 over-expressing tumors induce a higher frequency of apoptotic CD8^+^ T cells; cells which have been shown in several human and animal studies to be necessary for inhibition of tumor progression and metastasis [20], [21]. To our knowledge, this is the first evidence revealing that the regulation of COX2 can have a direct effect on bone-specific metastasis. Furthermore, subsequent PGE2 expression, can influence the recruitment of Tregs, and can explain the observed increased of CD8+ T cell apoptosis in the COX2 expressing primary tumors. Others, such as work by the Ostrand-Rosenberg or the Fulton group have shown myeloid derived suppressor cells (MDSCs) or NK cells, respectively, as the primary immune-suppressor recruited to the tumor to promote metastasis [22]. We believe that variability in the immune profile at the primary tumor is strongly correlated to the heterogeneous properties of the tumors themselves; though any immune suppression would be detrimental to tumor control. Combined with the published data of the negative side effects of anti-COX2 treatments, and the importance of inhibiting a pro-tumor immune tolerance, these data suggest that the treatment of breast cancer should be focused on targeting PGE2-specific receptors.

## Materials and Methods

### Animal model and cell lines

**Figure 1:**
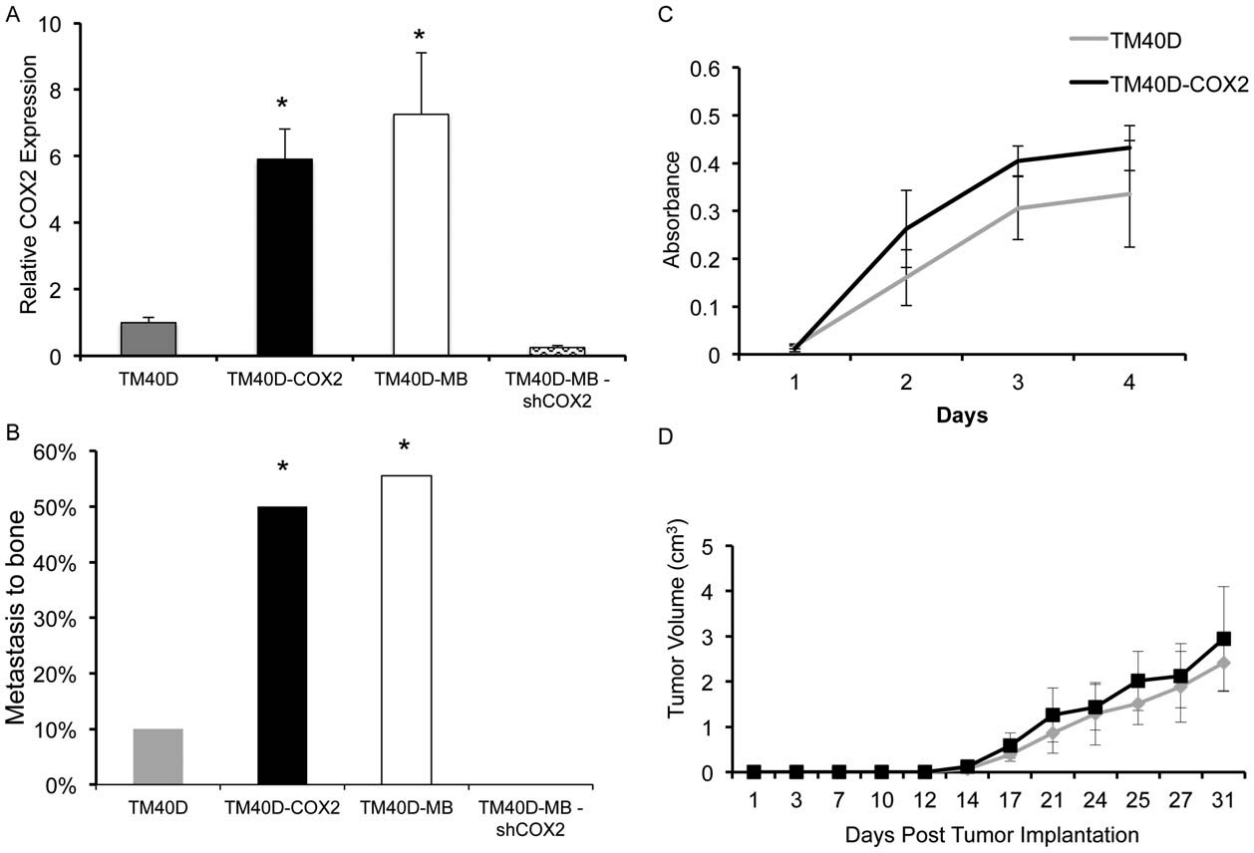
Breast cancer rate of metastasis to bone is not correlated to *in vitro* or *in vivo* growth rate. A. COX2 expression, measured by quantitative RT-PCR, by TM40D, TM40D-MB, TM40D-COX2, and TM40D-MB-shCOX2 cells. B. Metastasis to bone from primary TM40D, TM40D-COX2, highly aggressive TM40D-MB, or TM40D-MB-shCOX2 cells which also over-express COX2 was assessed at maximum tumor size by homogenizing both hind leg bones followed by in vitro growth under selection media and complemented with genomic DNA PCR for GFP. C. MTT cell proliferation assay performed on TM40D and TM40D-COX2 cells to measure *in vitro* growth. D. Mice were implanted with either low tumorigenic/COX2 expressing TM40D cells or COX2 over-expressing TM40D-COX2 cells into fourth mammary fat pad and monitored for *in vivo* tumor growth rates. N = 1/10, 4/8, 5/9, and 0/5 for TM40D, TM40D-COX2, TM40D-MB, and TM40D-MB-shCOX2, respectively. *p<0.05 compared to TM40D and TM40D-MB-shCOX2.

All experiments were done in accordance with protocols approved by the CCM Committee on Animal Care (CAR) institutional IACUC and in accordance with AAALAC. Female mice approximately 8 weeks old were used for all experiments. TM40D mammary tumor cells were derived from the FSK4 mammary epithelial cell line established *in vitro* from normal mouse mammary gland [23]. TM40D-MB tumor cells were isolated from bone by antibiotic selection after intracardiac injection of TM40D cells, according to a modified method by Li et al. [24]. For the TM40D-COX2 cell line, a COX2 full-length cDNA clone was purchased from ATCC and was inserted into a mammalian expression vector that was directed by an elongation factor promoter (pEF-COX2). The COX2 expression vector and a pEF empty vector were transfected into TM40D cells individually, and a pool of stable clones were harvested after 3 weeks of selection under G418 at 0.6 µg/ml, and were named as TM40D-C and TM40D-COX2. COX2 over-expression in TM40D-COX2 cells was verified and compared with TM40D-C (termed TM40D henceforth) cells by RT-PCR analysis. COX2 was over-expressed 6.2 fold in TM40D-COX2 compared to TM40D-C cells as previously described [25]. To study the effects of COX2 down regulation in the highly bone-metastatic TM40D-MB cells, a retrovirus expressing a short-hairpin RNA inhibitor (shRNA) of COX2 was used to transduce the TM40D-MB cells (TM40D-MB-shCOX2). This resulted in significantly decreased expression of COX2 as compared to the TM40D-MB vector control and TM40D-COX2 cells (Figure 1A). BALB/c-derived mammary tumor cells of very low metastatic potential, termed TM40D, the high metastatic potential TM40D-MB (metastatic to bone), and the COX2 over-expressing TM40D-COX2 lines were orthotopically implanted into the mammary fat pads of BALB/c mice. Unlike the TM40D tumors, TM40D-MB tumors spontaneously metastasized to bone with a rate of 53% [25].

### Syngeneic mouse model of breast cancer bone metastasis

For all tumor experiments, mice were injected bilaterally into the 4^th^ mammary fat pads with 1×10^6^ tumor cells. Tumor volume measurements were taken every other day, and tumor volume was calculated using the formula: (length×width^2^)/2. Mice were euthanized when tumors reached 2.0 cm, the maximum size allowed according to AAALAC guidelines and the rules set by the IACUC. For detection of spontaneous bone metastasis, hind leg bones were harvested and homogenized by pulverization. Cells were subsequently allowed to grow *in vitro* under selection of G418. Cultured cells were further confirmed for tumor lineage by isolating genomic DNA PCR for GFP expression, an ectopic tumor marker that had been stably transfected into the tumor cells. As negative control and confirmation of tumor specificity, crushed femurs of naive animals were included in the PCR analysis.

### MTT assay

TM40D and TM40D-COX2 cells were seeded into 96-well plates at a density of 1×10^3^ cells per well and incubated in DMEM medium containing 5% FBS. Cells were incubated for 4 hours with MTT (Sigma Chemical Co., USA, 5 mg/ml). Then the supernatant was removed and DMSO was added. Absorbance at 570 nm (A570) and DMSO (SigmaChemical Co., USA) was measured with a microplate reader (Model 550, Bio-Rad, USA). Actual absorbance = absorbance of the experimental group – absorbance of DMSO.

### Fluorescence-activated cell sorting (FACS) analysis

At maximum tumor size, spleen and tumor were excised and homogenized to obtain single cell suspensions, and erythrocytes were lysed as previously described [26]. To test for immune cell recruitment in spleen and tumor, 2×10^6^ cells from each sample were pre-incubated with anti-CD16/CD32 (2.4G2, eBioscience) to avoid non-specific binding of antibodies to FcγR . Cells were stained with the following fluorophore-conjugated anti-mouse monoclonal antibodies: anti-CD4, anti-CD25, anti-FoxP3, anti-CD8α, anti-CD45R, anti-Ly6G, anti-Ly6C, anti-F4/80, or anti-CD11b (BD Biosciences). To exclude dead cells from analysis, cells were stained with LIVE/DEAD fixable violet blue (Invitrogen). Cells were sorted on a FACS Canto II (BD Biosciences) and analyzed using FlowJo software (Tree Star).

### Enrichment of GFP-FoxP3+ Tregs

Tregs were enriched from splenocytes of healthy, unchallenged 7 week-old wild-type BALB/c mice using the regulatory T cell isolation kit (MACS, Miltenyi Biotec) protocol provided and as previously described [27]. Briefly, to remove splenocytes of B cells and myeloid cells, splenocytes were incubated with biotin-B220 (BD Biosciences) or biotin-CD11b (BD Biosciences), respectively, followed by streptavidin microbead magnetic column depletion (MACS, Miltenyi Biotec). Enrichment of CD4+ cells isolated from splenocytes was confirmed by FACS. GFP-FoxP3+ Tregs were then isolated (>96% purity) using a Beckman Coulter MoFlo (Beckman Coulter, USA) and cells were processed as necessary.

### Immunohistochemistry (IHC) and Immunofluorescence (IF)

For each tumor group, 5 µm thick sections of formalin-fixed paraffin embedded mammary tumors (3 mice per group) at maximum tumor size were deparaffinized and rehydrated in graded alcohol. Antigen retrieval was done using 1× Target retrieval solution (DAKO) and incubated with BSA for 30 minutes at room temperature. Sections were then incubated either primary antibodies anti-mouse CD4 (Abcam), anti-mouse CD8 (Santa Cruz Biotechnology), anti-mouse FoxP3 (eBiosciences), or Cleaved Caspase-3 (Cell Signaling) overnight at 4°C. For IF, the slides were washed twice with PBS and incubated with Alexaflour-594 and Alexafluor-488 secondary antibodies (Invitrogen), and counterstained with DAPI. For IHC, the slides were washed with PBS and incubated for 15 minutes with 4′,6-diamidino-2-phenylindole (Sigma), then washed with PBS and mounted with antifade mounting medium. Images were acquired using Zeiss Axiovert microscope (Zeiss, Germany) and Axiovision Rel. 4.5 Analysis System (Zeiss, Germany).

### RNA extraction and RT-PCR

RNA was harvested from purified CD4+ FoxP3+ Tregs (Rneasy kit, Qiagen) and RT-PCR was performed on reverse-transcribed cDNA (Superscript II, Invitrogen) using primers for the murine *EP1*, *EP2*, *EP3*, and *EP4* genes and GAPDH was used as a control for relative levels of total RNA, as previously described [28]. The following primers were used to amplify mouse *EP1* gene: 5′- TAG TGT GCA ATA CGC TCA GCG -3′ and 3′- GAG GTG ACT GAA ACC ACT GTG GGA CCA AGG CTT CAG AGA G-5′, *EP2* gene: 5′- GTG GCC CTG GCT CCC GAA AGT C -3′ and 3′- GGC AAG GAG CAT ATG GCG AAG GTG -5′, *EP3* gene: 5′- GGC ACG TGG TGC TTC ATC -3′ and 3′- GGG ATC CAA GAT CTG GTT -5′, and *EP4* gene: 5′- CGT AGT ATT GTG CAA GTC GC -3′ and 3′- GGC GAT GAG TAA GAT GAC CA -5′. These result in bands of 553, 535, 416, and 720 bp, respectively. Primers for human GAPDH were used as described previously [26]. Amplified products were visualized on an agarose gel.

### Migration Assay

Tregs were separated from naïve mouse thymus and lymph nodes using PE-conjugated CD25 (BD Biosciences), biotinylated CD4, streptavidin and anti-PE magnetic MACS beads (Miltenyi Biotec) and a MACS LS column (Miltenyi Biotec). The purity of Tregs isolated was checked using FACS. For the migration assay, the upper chamber of 5 µm uncoated 96-well ChemoTX system (Neuro Probe, Gaithersburg, MD) was used. Isolated Tregs were resuspended in RPMI containing 2% serum at 10^6^/ml concentration and seeded in triplicates on the top well in 20 µl. The bottom well was loaded in triplicate with 29 µl of media plus 5% serum (negative control), conditioned medium obtained from TM40D, TM40D-COX2, or TM40D-MB cells, or TM40D-COX2 medium containing anti-PGE2. After 4 hour incubation at 37°C, cells that migrated into the lower chamber were fixed with 1% paraformaldahyde, imaged by microscope with attached camera (Leica) and quantified using Image J software.

### Statistical analysis

Data for tumor metastasis are combined for all tumor injected animals. Tumor data with less than 10 animals was due to animal exclusion prior to sacrifice and analysis. Data for all other results are representative experiment of all repeats. Results are expressed as the mean ± standard deviation. Student's *t* tests, chi-squared, log-rank or ANOVA tests were used to determine statistical significance. A value of *p*<0.05 was considered statistically significant. Data were analyzed using Excel (Microsoft) and InStat 3 (GraphPad).

## Results

### COX2 promotes tumor progression in a syngeneic orthotopic transplantation model of breast cancer

Previous work from our laboratory has shown that the highly metastatic TM40D-MB breast cancer cell line has greater than a 4-fold increase in COX2 expression [25]. Manipulation of the low-metastatic TM40D mammary cancer cell line to over-express COX2 (TM40D-COX2) resulted in an increase in PGE2 production and exacerbated bone degradation due to an increase in osteoclast formation, an effect which was abrogated by treatment of the TM40D-COX2 cells with the COX2 inhibitor NS-398 [25]. Additionally, levels of COX2 were similar between TM40D-COX2 and TM40D-MB, 6–7 times higher than TM40D, and 25 times higher than TM40D-MB-shCOX2 when compared by quantitative RT-PCR (Figure 1A). We therefore wanted to directly assess the effects of COX2-modulated tumor cells on tumor progression using our syngeneic mouse model of mammary cancer metastasis. To determine the presence of tumor cells in bone, we employed the use of antibiotic selection of pulverized *ex vivo* bone isolated from tumor-challenged mice, grown *in vitro* and followed by PCR analysis of the cultured cells to detect the presence of the green fluorescent protein (GFP), an ectopic tumor marker that had been stably transfected into the tumor cells. We observed nearly a five-fold increase in bone metastasis occurring from the primary TM40D-COX2 tumor (50%) compared to the bone metastasis occurring from the TM40D (control vector) tumor (11%) or TM40D-MB-shCOX2 (0%), validating the metastasis-promoting action of COX2 in our mammary tumors (**Figure 1B**, p<0.05). The metastasis to bone observed in the TM40D-COX2 was similar to the rate of metastasis occurring from the highly aggressive TM40D-MB line (56%). Importantly, metastasis to bone appeared to be directly associated with elevated levels of COX2, as analysis of the brain, lung, and liver did not show variation in metastatic rates between the mice challenged with TM40D versus TM40D-COX2 (data not shown). To address whether the increased rate of metastasis was attributed to differences in proliferation between TM40D and TM40D-COX2, we compared *in vitro* cell proliferation and *in vivo* tumor growth between the groups. For *in vitro* proliferation experiments TM40D and TM40D-COX2 cell proliferation was compared by MTT assay for 72 hours (**Figure 1C**). Additionally, for the *in vivo* tumor growth analysis, TM40D and TM40D-COX2 tumor cells were implanted into the mammary fat pads of BALB/c mice and monitored for tumor growth (**Figure 1D**). We subsequently assessed the differences in time between TM40D and TM40D-COX2 tumor growth to maximum tumor size. Both TM40D and TM40D-COX2 tumors became palpable in the second week, and were all sacrificed in the fifth week following challenge with no differences in growth patterns. These data reveal that the differences in metastatic potential between TM40D and TM40D-COX2 are not due to variations in proliferation *in vitro* or *in vivo*. This suggests that there may be differences in the tumor microenvironment that induce variation in metastatic potential.

### TM40D-COX2 tumors promote a suppressive immune profile compared to TM40D tumors

**Figure 2:**
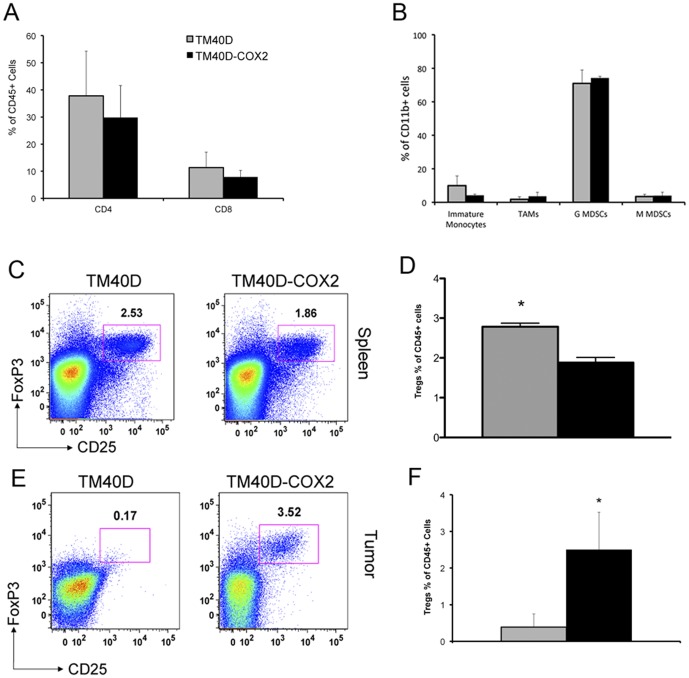
Tumor immune profile reveals elevated Tregs levels. TM40D (grey bar) or COX2 over-expressing TM40D-COX2 (black bar) cells were implanted in the fourth mammary pad of Balb/c mice. At maximum tumor volume tumors were assessed for (A) CD4+ and CD8+ T-cells, (B) immature monocytes (CD11b+ F4/80+ Ly6g+), tumor associate macrophages (CD11b+ F4/80+ Ly6g−), G MDSCs (CD11b+ Ly6c^low^ Ly6g+) and M MDSCs (CD11b+ Ly6C^hi^ Ly6g−). Tregs (FoxP3+CD4+ CD25+) were assessed via flow cytometry of spleens from TM40D and TM40D-COX2 challenged mice (C). Quantitative analysis of Tregs observed in the tumor of tumor challenged mice (D). Representative Treg levels in the primary tumor of mice challenged with TM40D or TM40D-COX2 mammary tumor cells (E). Quantitative analysis of Tregs observed in the spleen of TM40D versus TM40D-COX2 challenged mice (F). p<0.05 compared to TM40D group, n = 4–5 animals per group. *p<0.05 compared to TM40D.

Several recent studies have demonstrated a strong correlation between tumor metastasis and elevated levels of Tregs in various cancers, including breast [16], [29]–[32]. Cytokines, chemokines, and other factors such as COX2 can potentiate Treg recruitment or differentiation from naïve CD4+ cells. For our subsequent experiments, at the experimental endpoint, spleen and primary tumor were isolated and homogenized into a cell suspension for flow cytometry analysis. The data revealed that neither splenic (data not shown) or tumor CD4+ T cells (yielding about 35% of the immune profile) and CD8+ T cells (yielding about 10%) were altered due to COX2 expression (**Figure 2A**). Due to potential immune suppression by other cells, such as MDSCs, we characterized the tumor infiltration of myeloid-derived cells. Specifically, no differential recruitment of immature monocytes (CD11b+ F4/80+ Ly6G+), tumor associate macrophages (TAMs) (CD11b+ F4/80+ Ly6G−), granulocytic-myeloid derived suppressor cells (G-MDSCs) (CD11b+ Ly6C^low^ Ly6G+), or monocytic-MDSCs (M-MDSCs) (CD11b+ Ly6C^hi^ Ly6G−) in the primary tumor or the spleen were detected (**Figure 2B**, and data not shown, respectively). Over-expression of COX2 in the primary tumor was found to significantly attenuate splenic levels of Tregs by nearly 30% in mice challenged with TM40D-COX2 tumors, compared to mice with TM40D tumors (**Figure 2C and D**, *p*<0.05). In contrast, increase tumor densities of Tregs, measured by surface markers CD4 and CD25 plus intracellular FoxP3, as compared to TM40D control (**Figure 2E and F**), suggestive of a shift of the Treg population from the circulation to the primary tumor. Thus, modulation of tumor COX2 expression dramatically alters the recruitment of suppressive Tregs to both primary tumor sites.

**Figure 3:**
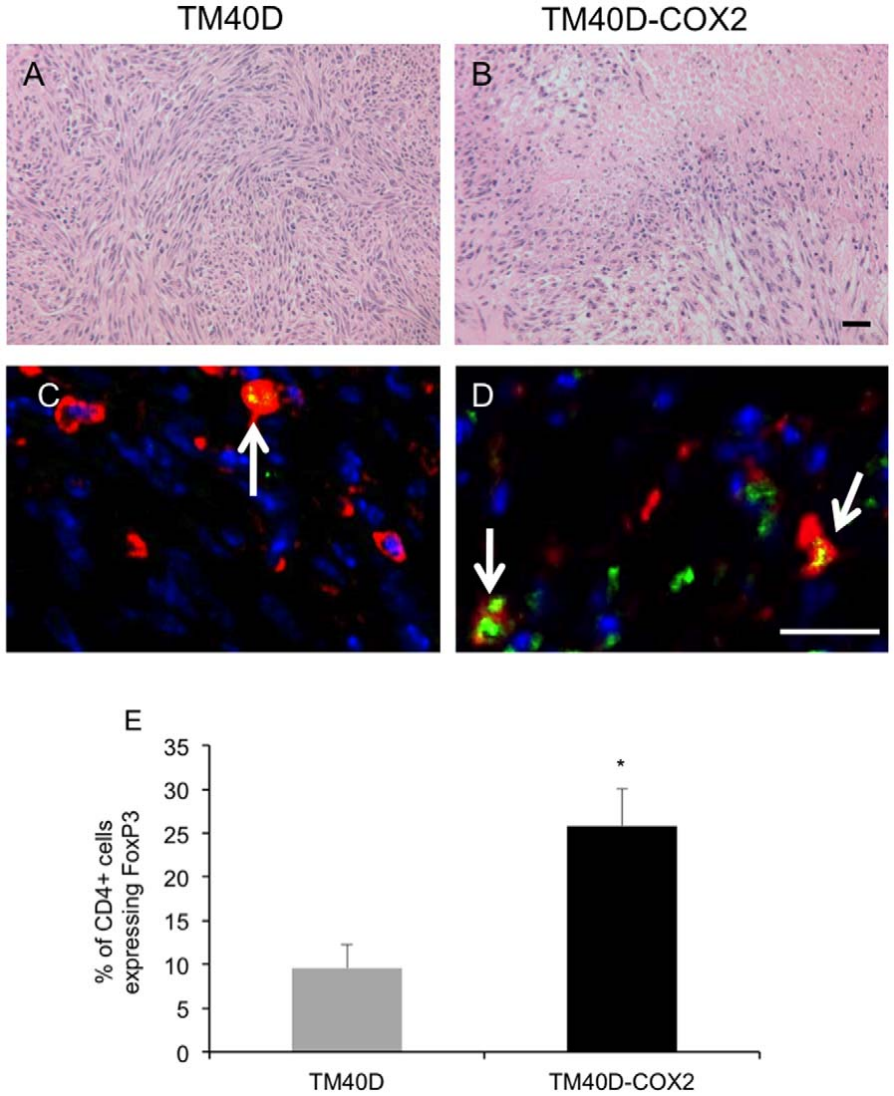
TM40D-COX2 tumors preferentially recruit Tregs to the tumor. Histological sections of TM40D and TM40D-COX2 tumors were stained with (A and B) H&E or (C and D) AlexaFluor 594 for CD4 (RED) AlexaFluor 488 for FoxP3 (GREEN), or Dapi for nucleus (BLUE). Arrows in panels C and D represent Tregs. E. Quantitative analysis of panels C and D measuring the percent of CD4+ cells that have FoxP3 co-localized to the cell. N = 3 samples per group, 8–10 sections per sample. *p<0.01 compared to TM40D. Bar = 20 µm.

In the tumor microenvironment, these findings were validated and confirmed by immunohistochemistry. Histological sections stained with H&E showed a varied profile between the TM40D and TM40D-COX2 tumors (**Figure 3A and 3B**, respectively). Specifically, the TM40D-COX2 tumors showed increased heterogeneity, with regions of exacerbated necrotic regions in the COX2-overexpressing tumors. To validate our FACS data, fluorescent immunostaining was performed on tumor sections. Sections were stained with anti-CD4 (red) and anti-FoxP3 (green) Tregs (arrows) in the TM40D and TM40D-COX2 tumors (**Figure 3C and 3D**, respectively). Analysis of the fluorescent immunohistochemistry showed the Tregs had infiltrated the TM40D-COX2 tumors at a significantly higher rate compared to the TM40D tumors (**Figure 3E**, p<0.05). These data suggest there is a means by which the over-expression of PGE2 can induce Treg recruitment from the circulation to the primary tumor.

### Tregs expressing the PGE2 receptors EP2 and EP4 are preferentially recruited to factors expressed by TM40D-COX2 tumor cells

**Figure 4:**
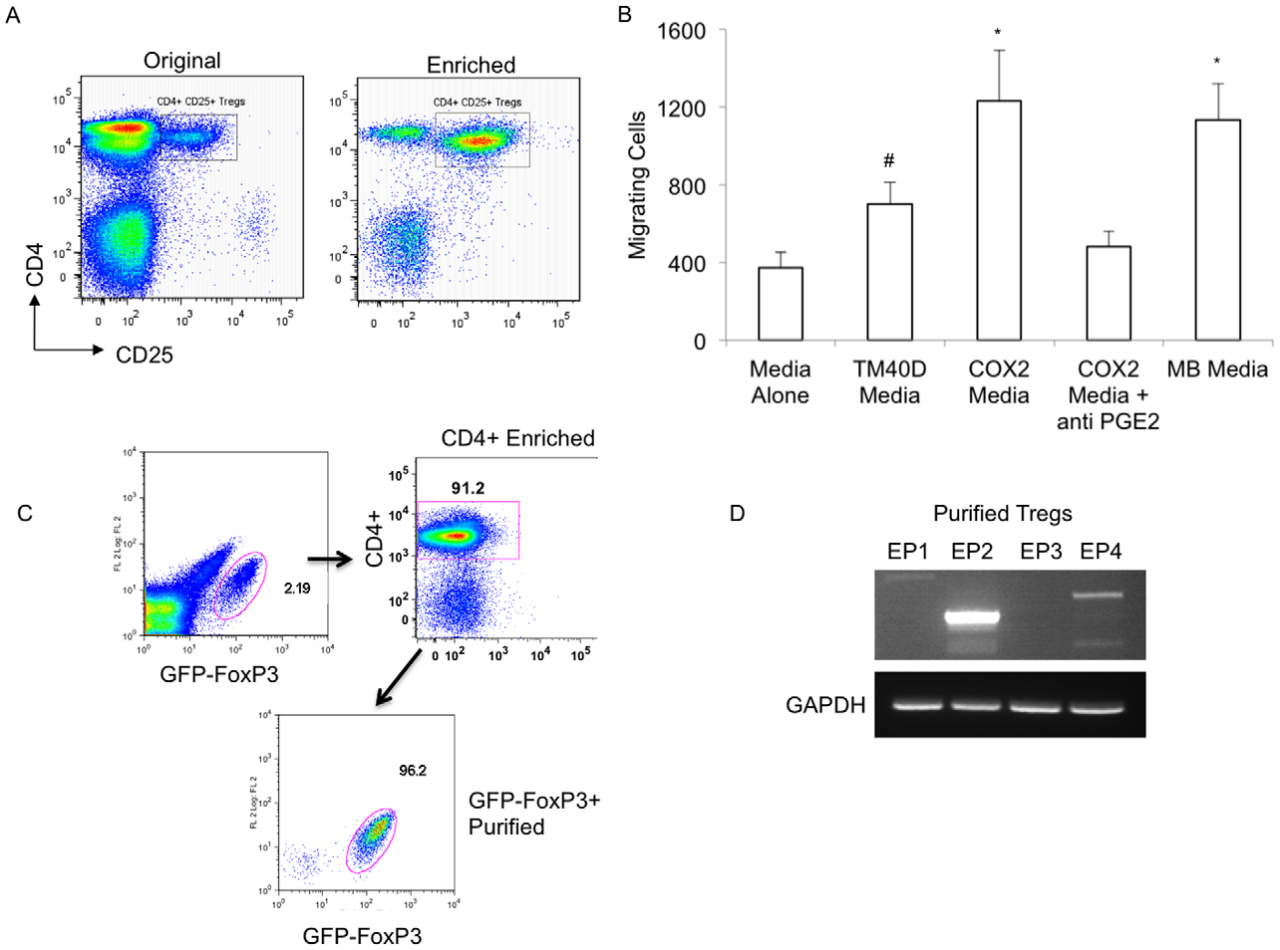
PGE2 directly involved in inducing Treg migration. CD4+ CD25+ Tregs were enriched by magnetic column (MACS) from naïve thymus and lymph nodes (A). Treg migration was measured using media alone, cell-free supernatant collected from TM40D, TM40D-COX2, or TM40D-MB cells (B). Additionally, the role of PGE2 was assessed by anti-PGE2 blocking antibody added to TM40D-COX2 media. Purified GFP-FoxP3+ cells were isolated from spleen following CD4+ T cell enrichment (C) and analyzed for expression of the PGE2 receptors EP1, EP2, EP3, or EP4 via RT-PCR with GAPDH as a control (D). #p<0.05, *p<0.01 compared to Media alone, N = 3 samples per group.

With increased Treg recruitment to the tumors over-expressing PGE2, we sought to gain an understanding of whether the increased frequency of Tregs was due to the direct recruitment of natural Tregs (nTregs), explaining the loss of Tregs observed in the circulation (**Figure 2E and F**) or the *de novo* conversion of CD4+ T cells into inducible Tregs (iTregs) in the TM40D-COX2 tumors. In order to assess whether Tregs can be recruited to the tumor by factors released by the TM40D-COX2 cells, CD4+ CD25+ Tregs were enriched by magnetic column (MACS) from naïve thymus and lymph nodes (**Figure 4A**). Subsequently, the rate of migration into cell-free supernatants from control media or harvested supernatant from TM40D, TM40D-COX2, or TM40D-MB cultures was measured (**Figure 4B**). Frequencies of migrated Tregs were found significantly elevated in the TM40D-COX2 and the TM40D-MB group, as compared to the TM40D group (p<0.01), which itself was elevated compared to control media (p<0.05). Furthermore, addition of anti-PGE2 to TM40D-COX2 media completely ablated the chemotactic potential observed with the TM40D-COX2 media alone, reducing the number of migrating Tregs to the level observed with the TM40D media (**Figure 4B**). The ability to inhibit the preferential migration of Tregs to the TM40D-COX2 media via an antibody to PGE2 suggests that these Tregs have PGE2 receptors. Splenocytes isolated from naïve BALB/c mice were isolated and depleted of CD11b+ myeloid cells by magnetic bead separation. Specifically, enriched CD4+ lymphocytes were positively selected and GFP-FoxP3+ Tregs were purified by FACS resulting in populations of >96% purity (**Figure 4C**). The Treg RNA was isolated and RT-PCR of the PGE2 receptors, EP1-4, was performed (**Figure 4D**). Similar to the naive CD4+ T cell parent previously published, CD4+ FoxP3+ Tregs retain a high level expression of EP2 and a lower level expression of EP4. PGE2 receptors EP1 and EP3 were not expressed in the Tregs. Thus, modulation of COX2 expression in these tumor cells has a direct effect on their ability to recruit Tregs, potential suppressors of the immune response.

### Increased Tregs in COX2-expressing TM40D tumors are paralleled by elevated of apoptotic CD8+ cells

**Figure 5:**
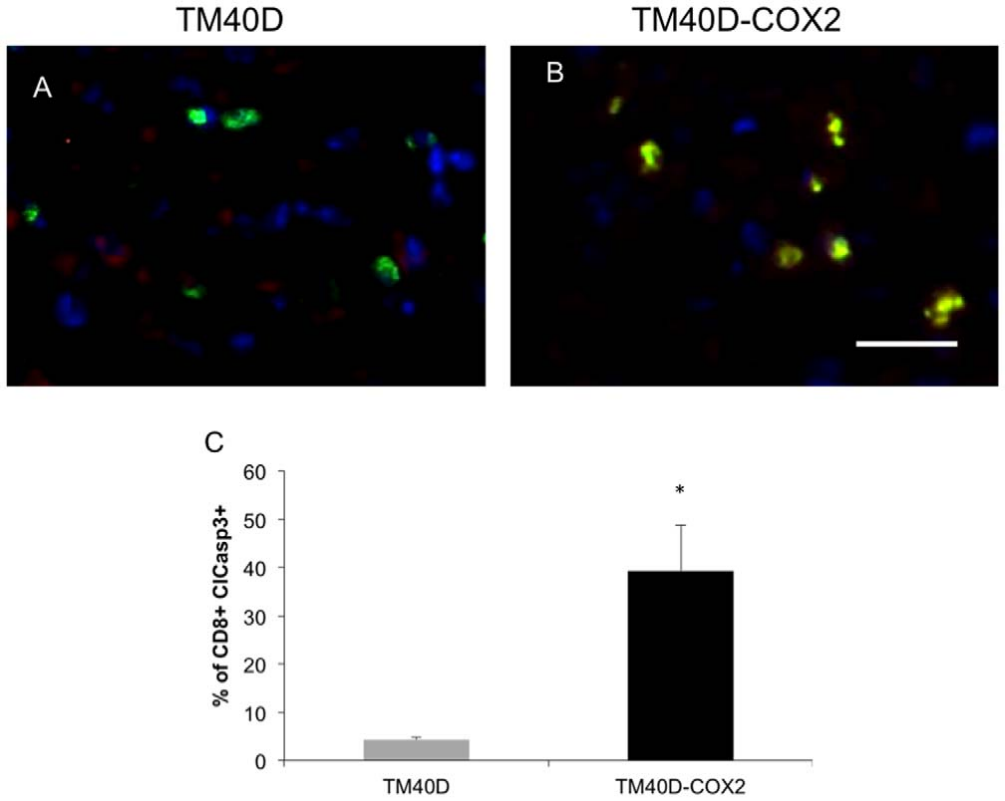
TM40D-COX2 tumors have increased apoptotic CD8+ T cells. Histological sections of TM40D and TM40D-COX2 tumors were stained with AlexaFluor 594 for CD8 (RED) AlexaFluor 488 for Cleaved Caspase 3 (ClCasp3+) (GREEN), and Dapi for nucleus (BLUE) (A and B, respectively). C. Quantitative analysis of panels A and B measuring the percent of CD8+ cells that have cleaved Caspase 3 co-localized to the cell. N = 3 samples per group, 8–10 sections per sample. *p<0.01 compared to TM40D.

Once recruited, the potential role of Tregs and their subsequent immune-suppressive capabilities were assessed. As previously reported, Tregs have the potential to exercise their immunosuppressive function by suppressing the immune response of many cells [33]. Additionally, Tregs have been shown to induce CD8+ T cell apoptosis [34]. Therefore, via immunofluorescence staining of TM40D and TM40D-COX2 tumors, we determined whether COX2 modulation would affect tumor CD8^+^ T cell function by measuring for apoptosis (**Figure 5A and 5B**). Analysis of CD8^+^ T cells (red), with co-localization of cleaved caspase-3 (green) revealed that the tumor density of CD8^+^ T cells undergoing apoptosis, measured as the percent of CD8+ cells that are cleaved caspase-3+, was significantly higher in the COX2-overexpressing TM40D-COX2 group compared to the TM40D tumors (**Figure 5C**, p<0.01). Interestingly, analysis of these tumors revealed regional distribution of apoptotic CD8+ cells in the tumor, showing sections of CD8 T cells as either all caspase 3+ or all caspase 3- in the COX2-overexpressing tumors. This may be attributed to the heterogeneous morphology and increased necrotic regions in the TM40D-COX2 tumors. Confirmed here, and as reported in Figure 2, there were no differences in total levels of CD8+ T cells and a small increase in total apoptotic cells, which can be attributed to the increase in apoptotic CD8+ cells in TM40D-COX2 versus TM40D tumors (data not shown). Thus, exacerbated tumor COX2 expression induces significant CD8^+^ T cell apoptosis, thereby disabling a potent mediator of antitumor immunity.

## Discussion

Increased COX2 or Treg accumulation in primary breast tumor is correlated, both clinically and in laboratory models, to increased metastasis. Additionally, clinical studies of lung, neck, gastric, and breast cancer patients have shown a correlation between increased COX2 expression with high Treg recruitment [18], [35]–[37]. These findings are often coupled to enhanced tumor progression in the patients with high COX2 and Tregs. Until now, there has not been any study showing a direct link between the increased Tregs in aggressive breast tumors and elevated COX2 expression. In our model of breast cancer metastasis we detected a significant up-regulation of COX2 in the highly metastatic TM40D-MB cells, as compared to the low-metastatic TM40D cells. We hypothesized that tumors may acquire a selective advantage by up-regulating expression of COX2; thereby evading T-cell mediated immune surveillance and promoting metastatic cancer progression. By developing a COX2 over-expressing mammary cancer cell line (TM40D-COX2), we now provide evidence that metastasis can be exacerbated through variable COX2/PGE2 expression, potentially explained by a PGE2-induced Treg recruitment to the tumor, and subsequent induction of CD8+ T cell apoptosis. Furthermore, elevated expression of COX2 (6–7 fold increase compared to TM40D-vector control) was sufficient to target metastasis to bone, as metastasis to brain, lung, and liver were not variable. In contrast the bone-specific metastasis was ablated following suppression of COX2 expression in the highly metastatic TM40D-MB cells (TM40D-MB-shCOX2). To our knowledge, this is the first study to show direct evidence that COX2 over-expression, and subsequent PGE2 production, promotes direct Treg recruitment and controls bone-specific metastasis.

COX2 expression has been linked to cancer progression due to its role in facilitating pro-angiogenic gene expression and angiogenesis, enhancing cell proliferation, inducing aromatase expression, and depressing the immune system [19], [38], [39]. Herein, we provide evidence that COX2, and subsequent PGE2 over-expression, results in a tumor environment that promotes Treg recruitment and attenuation of the normal immune response. The effect on Treg immune suppression was not attributed to differences in tumor burden, as all mice were euthanized at the same tumor volume. Furthermore, over-expression of COX2 in TM40D-COX2 cells had no effect on primary tumor growth *in vivo* or *in vitro*, but mice implanted with these tumors demonstrated significantly increased tumor levels of Tregs and a significant increase in spontaneous bone metastasis. Additionally, due to the similar *in vivo* tumor growth it is unlikely that COX2 is increasing tumor resistance to apoptosis, as other have shown. It is important to note that we did not observe any differences in vascularity when comparing histological sections of the tumors (data not shown), suggesting COX2 is working through mechanisms not involving angiogenesis or cell proliferation. Other mechanisms of action may include increased Treg migration from the circulation or Treg differentiation in the tumor from CD4+ T cells. These results point to the detrimental effect of COX2 expression by tumor in promoting Treg-mediated antitumor immunity.

In murine models, depletion of CD4+CD25+ T cells significantly augments the efficacy of cancer vaccination, underscoring the role of these cells in suppressing the immune responses against cancer cells [40], [41]. Herein, we show that Tregs are recruited to low-metastatic TM40D tumors and preferentially recruited to highly metastatic TM40D-COX2 tumors over-expressing COX2, and subsequently PGE2. We provide evidence that support a PGE2-specific recruitment of Tregs to the tumor from the circulation. Primary CD4+CD25+ T cells isolated from mouse thymus and lymph nodes preferentially migrate to media containing higher levels of PGE2, which is ablated with a PGE2-specific antibody. The response to PGE2 is not surprising as Tregs express both the EP2 and EP4 receptors. The EP receptors are known G protein-coupled receptors; EP2 and EP4 are stimulatory GαS receptors, EP1 is a GαQ receptor, and EP3 is a GαI/O receptor. Although many manuscripts have published that all receptors have been associated with inducing and inhibiting tumor and immune cell migration depending on the cell stimulated, the use of receptor antagonists to EP2 and EP4 have been shown to inhibit breast cancer progression [42]. Furthermore, induction of the EP1 receptor has been shown to also suppress breast cancer metastasis, suggesting the inhibition of the stimulating PGE2 receptors or induction of the inhibitory PGE2 receptor can attenuate breast cancer progression [43]. Alternatively, Treg accumulation in a tumor could be due to differentiation from naïve T cells from the periphery. A study by Yuan et al. showed that in the gastric cancer microenvironment, PGE2 could induce FoxP3 expression independently of TGF-β and IL-10 [44]. Additionally, others have shown that the PGE2-induced Treg conversion from naïve CD4+ cells requires the EP receptors. Specifically, *FoxP3* expression was significantly reduced in the absence of the EP4 receptor and ablated in the absence of the EP2 receptor expression following exposure to PGE2 [18]. Although studies have shown that PGE2 alone can directly induce FoxP3 expression [18], we believe there are many mechanisms, including directly recruiting Tregs to the tumor, which can ultimately manipulate the immune system to promote an immune-suppressive environment.

Our study provides evidence that increased Treg recruitment to the tumor is correlated with increased levels of CD8+ cells undergoing apoptosis. Previous studies have revealed that exposure to PGE2 induced prevention of direct and cross priming of antitumor CD8+ T cell responses to tumor cells *in vivo* [45]. We extend our understanding of the detrimental role PGE2 plays in the immune response by showing CD8+ T cells undergo increased level of caspase 3 cleavage in tumors expressing high levels of COX2. We believe this may be a direct effect of the recruitment of Tregs to the tumor, as Tregs have been shown to promote CD8+ T cell apoptosis. Further investigation into cytokine production is warranted for a complete understanding of the mechanisms of the Treg-CD8 T cell interaction [34]. Not surprisingly, we observed the increased CD8+ cleaved caspase 3+ cells in specific localized lymphoid-rich sections of the tumor, rather than a diffuse localization. This has been observed by others, including a recent publication which describes Tregs, and the effects of Tregs, at sites of lymphoid aggregates in breast tumors [46]. Due to published data having attributed increased Treg accumulation to blocking CD4/CD8 T cell, NK cell, and DC-mediated immune response (for review see [33], we believe targeting upstream of Treg recruitment would result in beneficial response to breast cancer progression.

COX2 has been shown to play an important role in influencing other immune suppressive cells during tumor progression. Specifically, COX2-induced PGE2 expression has been shown to promote MDSC accumulation in human and rodent cancers [47], [48]. Additionally, studies by the laboratory of Ostrand-Rosenberg have shown that PGE2 induce the differentiation of MDSCs from the bone marrow stem cells and receptor antagonist block the differentiation [22]. Furthermore, mice with EP2 knockout inoculated with 4T1 mammary carcinoma displayed delayed tumor growth and reduced numbers of MDSC compared to wild-type mice. Though our data does not show differences in the numbers of either monocytic or granulocytic MDSCs in response to the varied levels of COX2/PGE2 expression, we cannot comment on the activation state of the MDSCs residing in their respective tumor environments. Alternatively, increased COX2/PGE2 expression in breast cancer can also influence the activation state of immune cells. For example, breast cancer patients with elevated levels of COX2 and PGE2 within the tumor and the circulation presented T cells with decreased proliferation in response to CD3 antibody stimulation, reduced levels of TNF-α, interleukin (IL)-12, IL-2, and increased levels of IL-10 and IL-4. Additionally, dendritic cells from these patients showed significantly reduced B7 and CD40 expression as well as attenuated phagocytic ability [37]. These findings, coupled with our current data, suggest there are numerous pathways through which COX2/PGE2 can influence the immune profile of breast cancer. An explanation for these alternative consequences may have to do with the influence PGE2 has on the cytokine, chemokine, and growth factor production it is inducing in both the tumor cells as well as the recruited immune cells. Therefore, targeting the source of the initial immune-editing, specifically PGE2, would be encouraged to bypass the various downstream results of elevated COX2 expression.

Targeting COX2 as the therapeutic means of treating breast cancer has been the focus of both clinical and laboratory investigations. Recently, many findings have suggested that the inhibition of COX2 promotes many undesirable cardiovascular and gastrointestinal side effects to patients, even taken on a short-term basis. This may be due, in part, to inhibition in not only the production of prostaglandin E2, but also be a loss in the synthesis of prostaglandin D2 and F2R, as well as the cardio-protective prostacyclin (PGI2) and thromboxane A2, upon suppressing COX2 [49]. Specifically targeting one of the many downstream effectors of COX2, suggest a potential decrease in potential side effects when treating breast cancer patients, as well as others taking COX2 inhibitors for non-cancer diseases. Due to the removal of primary tumors from most patients soon after diagnosis, the utilization of anti-EP receptor therapy would be a beneficial treatment in targeting metastatic breast tumor cells overexpressing COX2. First, it may prevent the establishment of the tumor cells to secondary sites by inhibiting the recruitment of immune-suppressive Tregs, therefore allowing the ‘normal’ robust immune response necessary to clear cancer cells. Second it could decrease morbidity involved with breast cancer metastasis to the bone, specifically osteolytic bone lesions following metastasis by suppressing the COX2/PGE2-mediated action on osteoblasts, ultimately leading to the inhibition of osteoclast function. Based on the current data, future studies will focus on utilizing antagonists that are not only specific to PGE2, but rather to specific sub-receptors of PGE2, specifically EP2 and EP4 receptors, to treat breast cancer progression.
